# Probiotic *Lactobacillus reuteri* has antifungal effects on oral *Candida* species *in vitro*

**DOI:** 10.1080/20002297.2016.1274582

**Published:** 2017-01-18

**Authors:** Mette Rose Jørgensen, Camilla Kragelund, Peter Østrup Jensen, Mette Kirstine Keller, Svante Twetman

**Affiliations:** ^a^ Department of Odontology, Faculty of Health and Medical Sciences, University of Copenhagen, Copenhagen, Denmark; ^b^ Department of Clinical Microbiology, Rigshospitalet, Copenhagen, Denmark

**Keywords:** Beneficial bacteria, yeast, candidiasis, growth inhibition, co-aggregation, antimycotic

## Abstract

**Background:** An alternative approach for managing *Candida* infections in the oral cavity by modulating the oral microbiota with probiotic bacteria has been proposed.

**Objective:** The aim was to investigate the antifungal potential of the probiotic bacterium *Lactobacillus reuteri* (DSM 17938 and ATCC PTA 5289) against six oral *Candida* species (*C. albicans, C. glabrata, C. krusei, C. tropicalis, C. dubliniensis*, and *C. parapsilosis*).

**Design:** The lactobacilli were tested for their ability to co-aggregate with and inhibit the growth of the yeasts assessed by spectrophotometry and the agar overlay inhibition assay. Additionally, the pH was evaluated with microsensors, and the production of hydrogen peroxide (H2O2) by the lactobacilli was verified.

**Results:** Both *L. reuteri* strains showed co-aggregation abilities with the yeasts. The lactobacilli almost completely inhibited the growth of *C. albicans* and *C. parapsilosis*, but did not affect *C. krusei*. Statistically significant differences in co-aggregation and growth inhibition capacities between the two *L. reuteri* strains were observed (p<0.001). The pH measurements suggested that *C. krusei* can resist the acids produced by the lactobacilli.

**Conclusions:** *L. reuteri* exhibited antifungal properties against five of the six most common oral Candida species. Further, the results reconfirms that the probiotic capacity of *L. reuteri* is strain specific.

*Candida* species are commensals in the oral cavity and part of the normal microbiota in 25–75% of healthy individuals [1]. Nevertheless, *Candida* species are opportunistic pathogens which under certain circumstances cause infections in the oral mucosa, termed oral candidiasis. Infections are primarily caused by *Candida albicans* that has the ability to switch between the blastospore form and the more invasive hyphae form. Other *Candida* species have been isolated from infected sites in the oral cavity, including *C. glabrata*, *C. krusei*, *C. tropicalis*, *C. dubliniensis*, and *C. parapsilosis* [2,3]. Currently, an increase in fungal resistance to antimycotic therapy causes concern, and as a consequence an alternative bio-ecological approach of fungal management has been proposed [4].

There is a growing interest in probiotic bacteria to prevent and combat oral diseases [5]. Probiotic bacteria are defined by the World Health Organization as ‘Live microorganisms that, when administered in adequate amounts, confer a health benefit on the host’ [6]. The most common probiotic genera *Bifidobacterium* and *Lactobacillus* are believed to act by competitive exclusion of pathogens from the oral mucosal adhesion sites, by competing for available nutrients, and by altering the mucosal immune host defence [7,8]. In addition, some probiotic bacteria produce acids from carbohydrate fermentation rendering a low pH, and some lactic acid bacteria produce hydrogen peroxide (H_2_O_2_) and bacteriocins which are harmful to pathogens [5]. The probiotic *Lactobacillus reuteri* has been shown to survive the passage through the ventricle and to colonize the human gastrointestinal tract transiently [9]. Under certain favourable conditions, *L. reuteri* produces a broad-spectrum antimicrobial substance, reuterin, assumed to inhibit DNA synthesis in pathogens in the surrounding environment [10]. In a randomized placebo-controlled clinical trial, the authors’ research group has recently reported a significant reduction of the oral *Candida* load in a group of frail elderly persons after probiotic intervention with *L. reuteri* DSM 17938 and ATCC PTA 5289 in a combined lozenge [11].

The ability of lactobacilli to co-aggregate with pathogens such as *Candida* is central in biofilm formation and is a desired feature of probiotics. Through co-aggregation, the lactobacilli may achieve an adequate mass and thereby have the ability to create a hostile micro-environment around the *Candida* species with high concentrations of substances such as acids, H_2_O_2_, bacteriocins, and so on that possibly inhibit the pathogens’ growth [12]. *In vitro* studies have investigated co-aggregation and growth inhibition of probiotic and pathogenic bacteria [12–14], with the focus being on caries- and periodontitis-associated microorganisms [13–15]. For *C. albicans*, probiotic interference has been demonstrated with probiotic lactobacilli, for example *L. rhamnosus* GR-1, *L. reuteri* RC-14, and *L. reuteri* ATCC PTA 5289 [16–18]. To the authors’ knowledge, only a few studies have looked into the antifungal effect of probiotic lactobacilli on non-*albicans Candida* species commonly found in the oral cavity [19–21]. The aim of this study was therefore to investigate the *in vitro* abilities of two strains of the probiotic *L*. *reuteri*, with demonstrated ability to reduce the *Candida* load *in vivo* in a vulnerable population and to co-aggregate with and inhibit the growth of six opportunistic pathogenic oral *Candida* species. Additionally, the pH, as an expression of acid production by the lactobacilli, was evaluated through agar plates with *Candida-Lactobacillus* co-cultures. Moreover, the ability of the lactobacilli to produce H_2_O_2_ was verified.

## Materials and methods

### Strains and culture conditions

Two strains of the probiotic *L*. *reuteri* (DSM 17938 and ATCC PTA 5289; Biogaia, Stockholm, Sweden) were used in this study. The *Candida* strains used were six laboratory reference strains from the Culture Collection, University of Gothenburg, Sweden: *C. albicans* CCUG 46390; *C. dubliniensis* CCUG 48722; *C. glabrata* CCUG 63819; *C. krusei* CCUG 56126; *C. parapsilosis* CCUG 56136, and *C. tropicalis* CCUG 47037. In addition, six clinically isolated strains from humans were included, generously provided by the Department of Clinical Microbiology, Rigshospitalet, Copenhagen, Denmark. Prior to the *in vitro* studies, the clinical strains were characterized by matrix-assisted laser desorption/ionization time-of-flight (MALDI-TOF) mass spectrometry [22] to confirm their identity as *C. albicans* CBS 562 NT, *C. dubliniensis* 41_3 ZZMK, *C. glabrata* CBS 863, *C. krusei* (*Issatchenkia orientalis* RV 491), *C. parapsilosis* 26 PBS, and *C. tropicalis* DSM 7524.

The lactobacilli were initially cultured on de Man Rogosa Sharpe (MRS) agar (Oxoid Ltd., Basingstoke, Hampshire, UK) for 24 h in an anaerobic chamber at 37°C (10% H_2_, 5% CO_2_, and 85% N_2_). The *Candida* strains were cultured on BD Difco™ Sabouraud Maltose (DSM) agar (Becton, Dickinson and Company, Sparks, MD) for 24 h in ambient air at 37°C.

### Co-aggregation assay

The co-aggregation was determined spectrophotometrically (Genesys™ 10S UV-Vis Spectrophotometer; Thermo Fisher Scientific, Waltham, MA) and executed as described previously by Collado et al. [12]. In brief, one distinct colony of overnight cultured lactobacilli was transferred to 5 mL MRS broth and incubated at 37°C for 24 h under anaerobic conditions. The *Candida* strains were similarly harvested and aerobically incubated in DSM broth at 37°C for 24 h. After incubation, the lactobacilli and *Candida* were harvested by centrifugation at 855 g (Sigma 2-6 Compact Centrifuge, Sigma, Poole, UK) for 10 min at room temperature, washed three times in phosphate-buffered saline (PBS), and resuspended in neutral 10 mmol/L PBS (pH 7.0). The absorbance was adjusted to an optical density (OD) of 0.5 at 600 nm (approximately 10^8^ cfu/mL of the lactobacilli) by using the spectrophotometer to ensure identical densities at baseline. Equal volumes (1.0 mL) of the lactobacilli and *Candida* strains were mixed and incubated aerobically at 37°C for 1, 2, and 4 h without agitation, but were vortexed prior to each OD measurement. Co-aggregation was calculated by using the equation [12,15]:

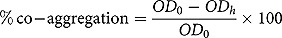

where OD_0_ is the absorbance of the mixed suspension at baseline (0 h) and OD_h_ is the absorbance of the mixed solutions at different time points (1, 2, and 4 h). The assays were carried out in duplicate and repeated four times on different occasions.

### Agar overlay interference test

The growth inhibition assay was performed as described earlier [14,23]. In brief, one distinct colony of overnight cultured lactobacilli was transferred to 5 mL MRS broth and incubated at 37°C for 24 h under anaerobic conditions. The following day, the lactobacilli were harvested by centrifugation at 855 g  for 10 min at room temperature. The supernatants of the two *Lactobacillus* strains were obtained after centrifugation and filter sterilized by the aid of sterile 20 mL syringes (Omnifix®; B. Braun, Melsungen, Germany) and syringe filters, 0.8/0.2 µm pore size membranes (Acrodisc® PF Syringe Filter; Pall Life Sciences, Lund, Sweden). The pellets were washed three times in PBS and resuspended in MRS broth. The OD was adjusted spectrophotometrically to 1.8 at 630 nm (corresponding to approximately 10^9^ cfu/mL). The cultures were then serially diluted in MRS broth in 10-fold steps. One millilitre of the supernatants, undiluted suspensions, and cell suspensions corresponding to approximately 10^7^, 10^5^, and 10^3^ CFU/mL were added to 24 m sterilized molten MRS agar (~45°C) in Petri dishes, and the agar was allowed to solidify. The plates were incubated overnight at 37°C under anaerobic conditions.

One single colony of each of the overnight cultured *Candida* strains was added to 5 mL DSM broth and aerobically incubated at 37°C for 24 h. The following day, one additional layer of 25 mL of molten sterile DSM agar was poured on top of the MRS agar with grown lactobacilli and supernatants, and was allowed to solidify and air-dry for 3 h at room temperature. The overnight-cultured *Candida* strains were diluted in DSM broth to a final OD of 0.2 at 500 nm. The *Candida* suspensions were stamped on the plates with a Steers steel-pin replicator (CMI-Promex ICN, Pedricktown, NJ) and left to dry for 1 h at room temperature. The plates were subsequently aerobically incubated overnight at 37°C. As controls, the *Candida* strains were also stamped on top of plates with no lactobacilli within the bottom MRS agar layer.

The assays were carried out in duplicate and repeated four times on different occasions. The results were evaluated in accordance with Simark-Mattson et al. [23] as follows: a score of 0 = complete inhibition (no visible colonies); a score of 1 = slight inhibition (at least one visible colony but definitely smaller amounts than at control plate); and a score of 2 = no inhibition (colonies equal to those at the control plate). Two observers scored the plates independently, and in case of disagreement, consensus was reached through discussion.

### Microsensor measurements of pH

To estimate the acid production of the lactobacilli and the effect of pH on *Candida* growth, pH was measured with a pH microelectrode (pH-25; Unisense A/S, Århus, Denmark) using a modification of a previously described procedure [24] in selected plates from the abovementioned agar overlay interference test. A reference electrode was used to establish a reference potential against the pH microelectrode (ref-100, Unisense A/S). The electrodes were mounted in a motorized PC-controlled profiling setup (MM33 and MC-232, Unisense A/S). Positioning and data acquisition were controlled by dedicated software (Sensortrace Pro 2.0, Unisense A/S). The pH and reference electrodes were calibrated with buffers of pH 4 and 7 at room temperature. The pH microelectrode had a detection limit of 0.1 pH units. The pH was measured on the final day of the agar overlay interference test after scoring the plates. Selection of the plates was based on the results from the interference test: pH was measured through dense, non-inhibited colonies of *Candida* (*C. krusei* CCUG 56126 and *C. tropicalis* CCUG 47037) and through vague, almost completely inhibited colonies (*C. albicans* CCUG 46390 and *C. glabrata* CCUG 63819) incubated on plates with *L. reuteri* DSM 17938 and ATCC PTA 5289 (10^3^ CFU/mL), respectively. In addition, pH was measured in control plates containing only the lactobacilli in the bottom agar layer, but with no *Candida* incubated, and in a control plate with *Candida* strains but without lactobacilli. Finally, pH was measured at a control agar plate without lactobacilli or *Candida* strains. Before commencing the measurements, the pH and reference electrodes were placed approximately 2 mm above the surface of the *Candida* colonies. The electrodes were set to move in steps of 100 µm, and pH was measured from the surface of the colonies and then for every 100 µm until the depth of 4.5 mm into the agar to make sure the sensors had reached the bottom agar layer containing the lactobacilli. The experiment was repeated twice at two different occasions.

### H_2_O_2_ production by the lactobacilli

In order to verify the ability of the two *L. reuteri* strains to produce H_2_O_2_, a method described by Kang et al. [25] was used. Briefly, one single colony of overnight cultured *L. reuteri* strains was plated onto MRS agar containing 0.25 mg/mL 3,3',5,5'-tetramethylbenzidine (Sigma, Poole, UK) and 0.01 mg/mL peroxidase from horseradish (Sigma) and incubated anaerobically for 48 h at 37°C. As controls, two bacterial strains known to produce H_2_O_2_ (*Streptococcus sanguinis* and *S*. *mitis*) and one strain known not to produce H_2_O_2_ (*S*. *salivarius)* [26] were grown on similar plates incubated under the same conditions. After incubation, the plates were exposed to ambient air, and colonies displaying a surrounding blue halo were considered to produce H_2_O_2_.

### Statistical analysis

Statistical analyses were processed with SAS Enterprise guide software v7.1 (SAS Institute, Inc., Cary, NC). A *p*-value of <0.05 was considered statistically significant. Data analysis on co-aggregation was subjected to one-way analysis of variance and Tukey’s *post-hoc* analysis, and presented as mean and standard deviations (*SD*). Data obtained from the agar overlay interference test were analysed by the chi-square test and presented as the median inhibition score.

## Results

### Co-aggregation between lactobacilli and yeasts

**Figure 1.**
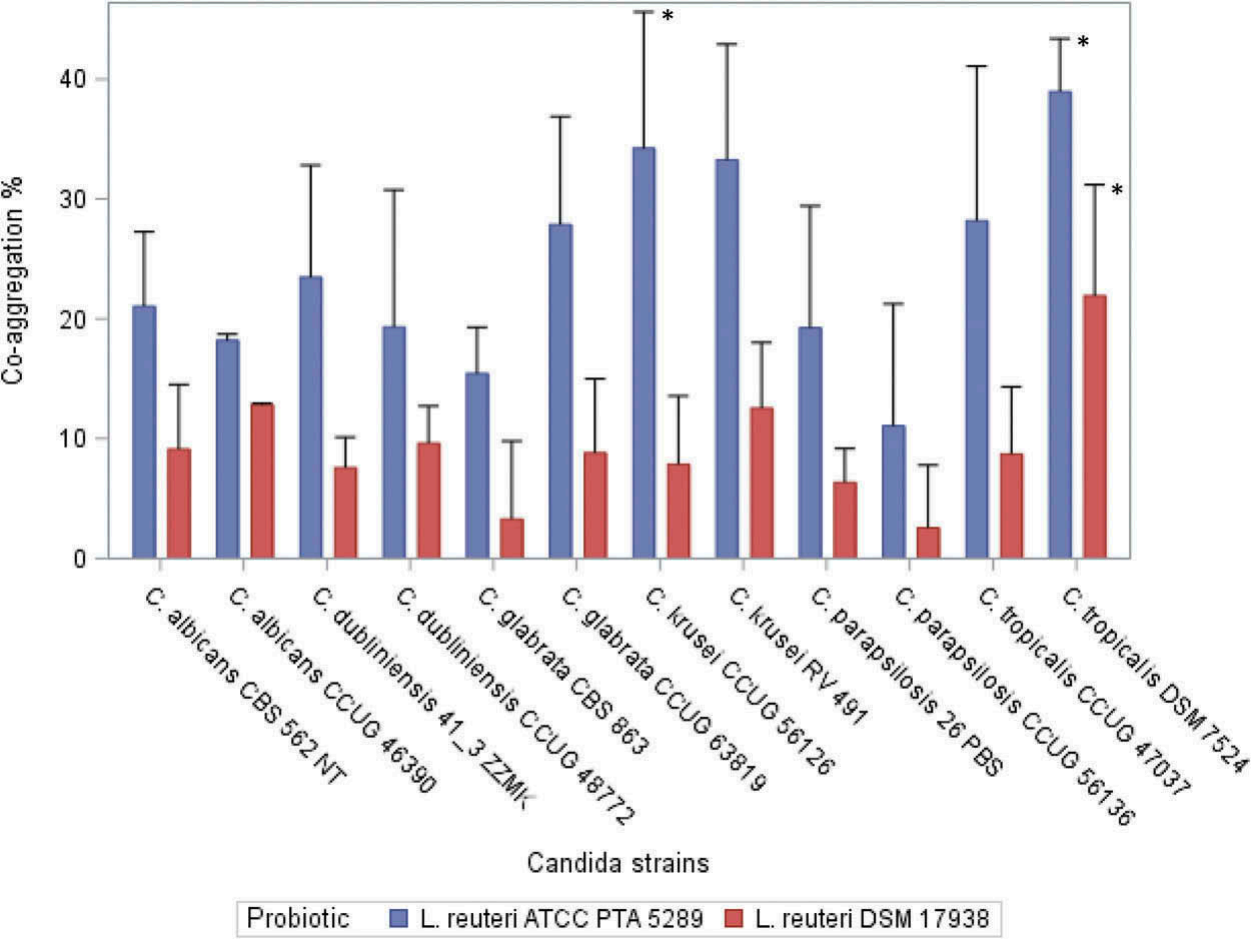
Co-aggregation ratio (%) between *Candida* strains and the *Lactobacillus reuteri* strains after 4 h of incubation. Mean, error bars indicate standard deviations. *Statistically significant differences (*p* < 0.05) .*L. reuteri* DSM 17938: *C. tropicalis* DSM 7524 showed significantly higher co-aggregation ability compared with *C. krusei* CCUG 56126, *C. dubliniensis* 41_3 ZZMK, *C. parapsilosis* 26 PBS, *C. glabrata* CBS 863, and *C. parapsilosis* CCUG 56136. *L. reuteri* ATCC PTA 5289: *C. tropicalis* DSM 7524 showed significantly higher co-aggregation ability compared with *C. glabrata* CBS 863 and *C. parapsilosis* CCUG 56136. *C. krusei* CCUG 56126 showed a significantly greater ability to co-aggregate compared with *C. parapsilosis* CCUG 56136.

*L. reuteri* ATCC PTA 5289 and *L. reuteri* DSM 17938 showed the ability to co-aggregate with both clinical and reference *Candida* strains. Co-aggregation increased significantly over time (1, 2, and 4 h) for all combinations of *Candida* and probiotic strains (*p* <0.05). For all the tested *Candida* strains, *L. reuteri* PTA 5289 showed a statistically significantly higher co-aggregation ratio than *L. reuteri* DSM 17938 after 4 h incubation (*p* <0.001). The results of the co-aggregation ratios (%) after 4 h are presented in Figure 1.

### Growth inhibition of Candida by L. reuteri

The results of the growth inhibition assays are summarized in Table 1. Both *L. reuteri* strains were able to inhibit the growth of five of the tested *Candida* strains but not the two *C. krusei* strains. In general, *L. reuteri* DSM 17938 was significantly better at inhibiting the growth of the *Candida* strains than *L. reuteri* PTA 5289 was (*p* <0.001); the latter showed no complete inhibition of any of the *Candida* strains. No statistically significant differences were found between the two *L. reuteri* strains for *C. krusei*. High cell concentrations (10^9^ cfu/mL) of *L. reuteri* displayed no superiority at inhibiting the *Candida* strains compared with lower cell concentrations of the bacteria (<10^7^ cfu/mL) in the bottom agar layer. For *C. tropicalis* CCUG 47037, an even weaker inhibition tendency was observed at high *L. reuteri* DSM 17938 cell concentrations compared with lower cell concentrations, whereas *C. glabrata* CBS 863 was completely inhibited at high *L. reuteri* DSM 17938 cell concentrations compared with slight inhibition at lower concentrations.

**Table 1.** Growth inhibition of six clinical isolates and six reference *Candida* strains by two strains of the probiotic *Lactobacillus reuteri* at different cell concentrations.

	*L. reuteri* ATCC PTA 5289			*L. reuteri* DSM 17938		
	CFU/mL					
*Candida* strain	10^9^	10^7^–10^5^	10^3^	10^9^	10^7^–10^5^	10^3^
*C. albicans* CBS 562 NT	1	1	1	0	0	0
*C. albicans* CCUG 46390	1	1	1	0	0	0
*C. dubliniensis* 41_3 ZZMK	1	1	1	1	0	1
*C. dubliniensis* CCUG 48722	1	1	1	1	1	1
*C. glabrata* CBS 863	1	1	1	0	1	1
*C. glabrata* CCUG 63819	1	1	1	1	1	1
*C. krusei* RV 491	2	2	2	2	2	2
*C. krusei* CCUG 56126	2	2	2	2	2	2
*C. parapsilosis* 26 PBS	1	1	1	1	0	0
*C. parapsilosis* CCUG 56136	1	1	1	1	1	1
*C. tropicalis* DSM 7524	1	1	1	1	1	1
*C. tropicalis* CCUG 47037	2	1	1	2	1	1

Inhibition scores according to Simark-Mattsson et al. [23]: 0 = complete inhibition (no visible colonies); 1 = slight inhibition (at least one visible colony but definitely smaller amounts than at the control plate); and 2 = no inhibition (colonies equal to those at the control plate).

### Bacterial acid production measured by ph

**Figure 2.**
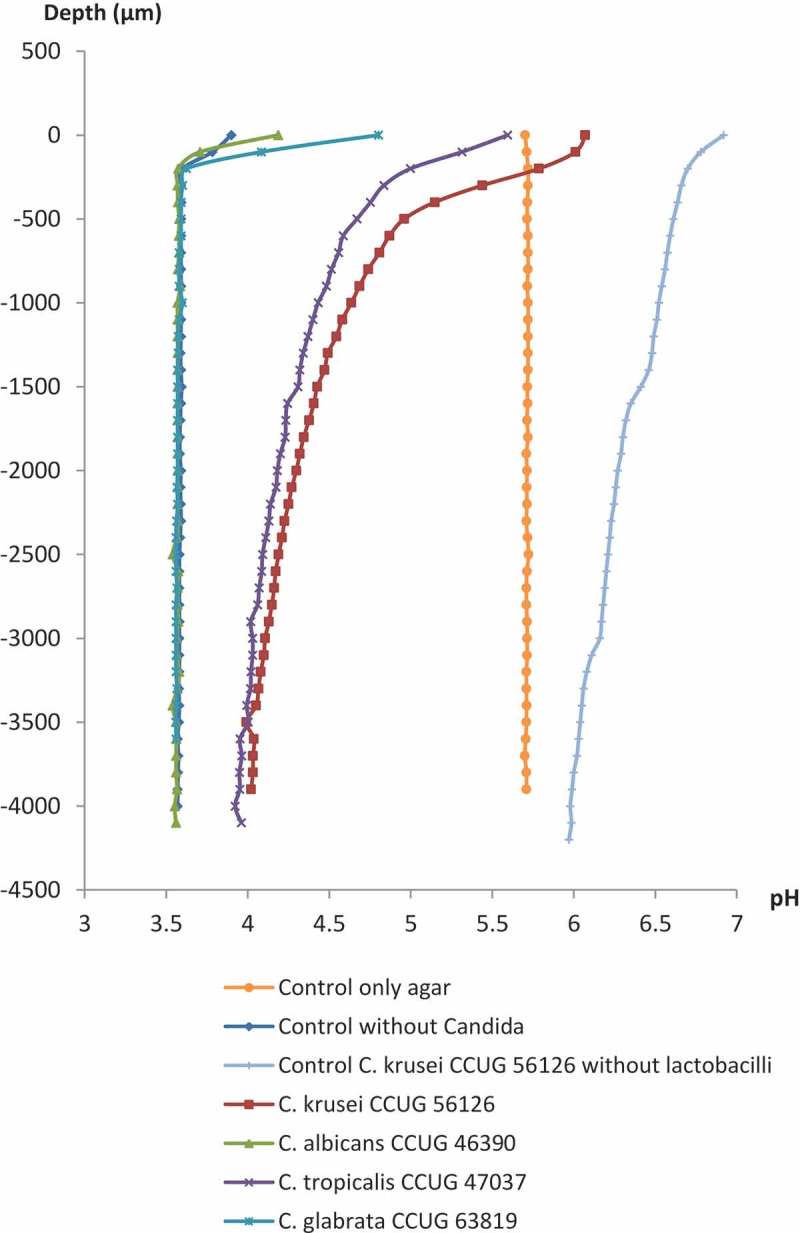
Microsensor measurement of pH at selected *Candida* strains grown by the agar overlay technique with *L. reuteri* ATCC PTA 5289 (10^3^ cfu/mL) in the bottom agar layer. Zero on the vertical axis represents the first reading when the sensors hit either a *Candida* colony or the agar plates without *Candida* colonies. For the *Candida* controls without lactobacilli, data are only presented for *C. krusei* CCUG 56126.

**Figure 3.**
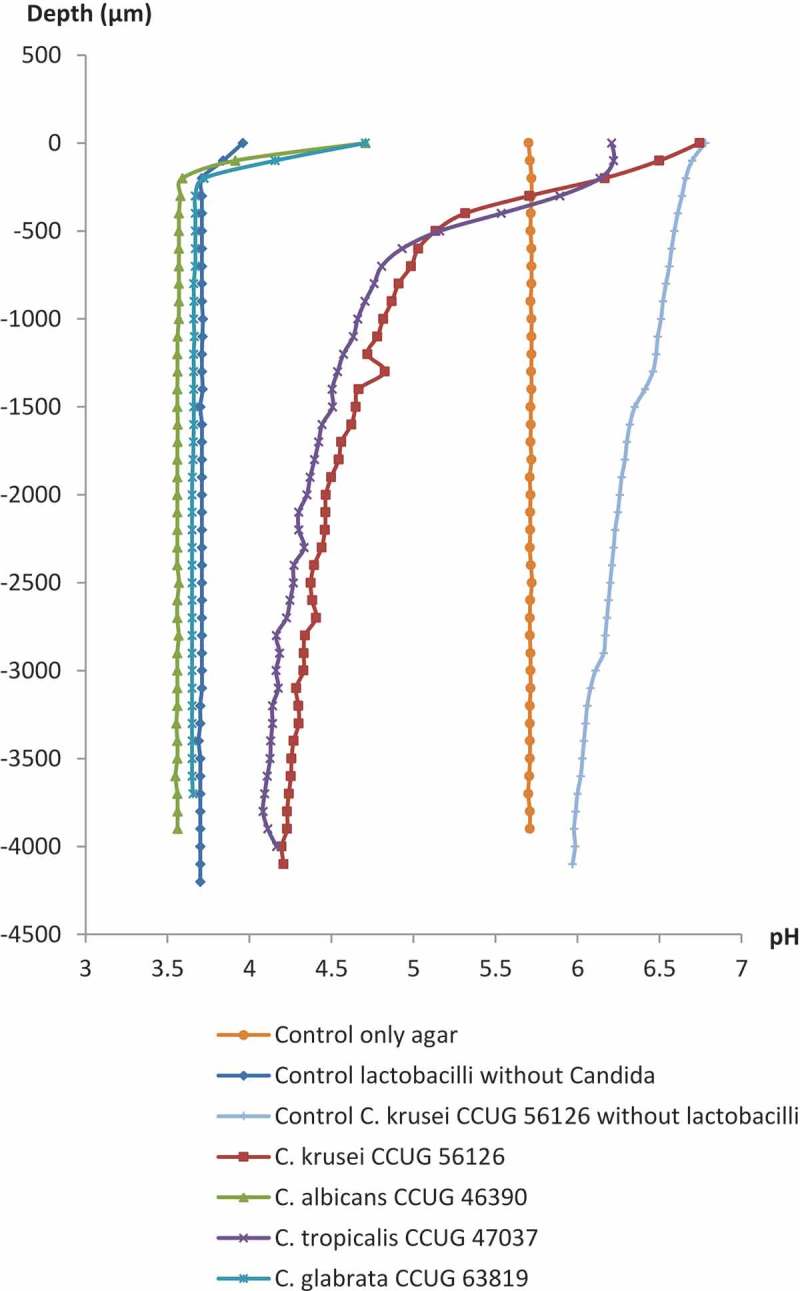
Microsensor measurement of pH at selected *Candida* strains grown by the agar overlay technique with *L. reuteri* DSM 17938 (10^3^ cfu/mL) in the bottom agar layer. Zero on the vertical axis represents the first reading when the sensors hit either a *Candida* colony or the agar plates without *Candida* colonies. For the *Candida* controls without lactobacilli, data are only presented for *C. krusei* CCUG 56126.

Typical results of the pH microsensor measurements in the agar overlay cultures of the lactobacilli and *Candida* strains and control plates are shown in Figures 2 and 3. Similar pH microprofiles appeared for both strains of the lactobacilli incubated with the *Candida*; pH measured through the dense colonies of the *Candida* species that were not inhibited by the lactobacilli at the inhibition assay (*C. krusei* CCUG 56126 and *Candida tropicalis* CCUG 47037) was approximately pH 6.0 at the surface of the colonies, remaining above pH 4.5 until a depth of 1,500 µm, and only slowly becoming more acidic throughout the agar layers approaching approximately pH 4.0 in the bottom agar layer. In contrast, the pH measured through the almost completely inhibited colonies of *C. albicans* CCUG 46390 and *C. glabrata* CCUG 63819 already reached a very acidic level of approximately pH 3.6 only a few 100 µm under the surface and remained stable throughout the agar layers.

### H_2_O_2_ production by the lactobacilli

Both *L. reuteri* DSM 17938 and *L. reuteri* PTA 5289 showed a positive reaction for H_2_O_2_ production by displaying clear-blue haloes around incubated colonies after around 1 h exposure to ambient air. A similar colour change was observed for *S. sanguinis* and *S. mitis* but not for *S. salivarius*, and the two *L. reuteri* strains were therefore verified as H_2_O_2_ producers.

## Discussion

To the authors’ knowledge, this is the first study to demonstrate that probiotic lactobacilli can hamper the growth of a selection of the most commonly isolated *Candida* species in the oral cavity under *in vitro* conditions. It was found that both *L. reuteri* strains were able to co-aggregate with the yeasts, displaying the highest co-aggregation ratios with *C. tropicalis* and *C. krusei*. Unfortunately, these two *Candida* species seemed less susceptible to the substances produced by the lactobacilli, as their growth was only slightly inhibited in the agar overlay inhibition assay. In an *in vitro* study by Verdenelli et al. [20], *L. plantarum* 319 displayed the highest degree of co-aggregation with *C. glabrata* and *C. albicans* among five *Lactobacillus* strains tested. As is the case for many probiotic properties of lactobacilli, co-aggregation also appears to be strain specific and is unique for each *Lactobacillus* strain involved. This was confirmed in this study, since *L. reuteri* ATCC PTA 5289 generally showed the highest co-aggregation capacity with all the *Candida* strains, and interestingly, this probiotic strain originates from the oral cavity. Co-aggregation is a recognized feature of the early biofilm formation and involves adhesion receptor interactions between complementary molecules on the microbial cell surfaces [27]. As such, probiotic competition with pathogenic *Candida* strains for binding sites in the oral biofilms may partly explain the beneficial outcome of previous clinical trials [5,11].

The agar overlay interference test is a well-proven and relatively simple technique for exploring the inhibition capabilities of probiotic bacteria against oral pathogens [14,18,23]. Moreover, the technique allows assessment of multiple *Candida* strains on a single plate and with different cell concentrations of the lactobacilli in the bottom agar layer. In this study, both *L. reuteri* strains were able to inhibit the growth of most of the *Candida* strains, except for *C. krusei* and to some extent *C. tropicalis*. This confirms the findings of other *in vitro* studies that investigated the antifungal effect of lactobacilli on *Candida* using varying techniques [16,19–21]. Jiang et al. [28] found that the most susceptible yeast to lactobacilli was *C. albicans*, whereas *C. krusei* was unaffected under all experimental conditions. Likewise, Zhao et al. [29] failed to inhibit *C. krusei* in a disc diffusion model. In this study, *L. reuteri* DSM 17938 demonstrated stronger inhibition ability than *L. reuteri* ATCC PTA 5289 did. The pH was measured through the agar layers after 48 h of incubation of the lactobacilli, and both *L. reuteri* strains were equally good at producing organic acids, resulting in a pH close to 3.6 in the agar. Hence, superior interference of *L. reuteri* DSM 17938 must likely be explained by other factors, such as an enhanced H_2_O_2_ or reuterin production which has been suggested in other studies [10,16,30].

It is likely that the observed inhibition of the yeasts is caused by the acidic environment in the agar due to lactic acid and other organic acids produced by the two *L. reuteri* strains, either directly or due to the production of bacteriocins at low pH [31]. Neutral-to-alkaline extracellular pH is considered an optimal pH for the growth of *Candida* species, since it induces hyphal morphogenesis in *C. albicans* [32], while at low pH, the fungus remains in the less virulent budding yeast form [33]. In this study, a very acidic environment was found in the agar only a few 100 µm under the surface of the almost completely inhibited colonies of *C. albicans* and *C. glabrata*. The un-dissociated form of lactic acid has been shown to pass the plasma membrane of yeast cells, leading to an increased activity of an energy-consuming plasma membrane H^+^-ATPase that removes protons from the intracellular environment. The increased H^+^-ATPase activity exhausts the available energy for growth and metabolism, leading to growth inhibition and finally cell death [31,34]. Moreover, in this study, a completely different result was observed for the non-inhibited *C. krusei* and to some extent *C. tropicalis*. Somehow, these fungi had neutralized the acids, creating a pH of almost 6.0 at the surface and only slowly decreasing down through the agar. This indicates that these fungi inhibit the acidification caused by the lactobacilli to ensure their survival. In experimental studies, Halm et al. [31] found *C. krusei* to be much more tolerant to lactic acid at low pH than *Saccharomyces cerevisiae*, since lactic acid only induced a weak short-term response in the intracellular pH of *C. krusei*. It is possible that *C. krusei* (i) actively produces extracellular ammonia (NH_3_) which will increase the surrounding pH [32,35], (ii) has less permeable plasma membranes to lactic acid, (iii) has a higher buffer capacity inside the cells, or (iv) has a higher H^+^-ATPase capacity than the other *Candida* species tested in this study [31]. A combination of the abovementioned factors may also occur.

Acid production by the lactobacilli may be a concern in regard to the development of new caries lesions in the oral cavity. Lactobacilli and streptococci, especially *S. mutans*, are abundant acidogenic species in caries lesions in the oral cavity [36]. Yet, *L. reuteri* strains have been shown to reduce the number of *S. mutans* in clinical trials after a continuous regular intake due to its production of substances with potent inhibitory activity on a wide range of bacterial species [37]. However, the species cannot colonize the oral cavity after ended intake and is therefore not considered a threat to the development of new carious lesions [38].

The strong oxidant H_2_O_2_ is a potent growth inhibitor and is mainly produced by oxidases in the carbon and energy metabolism of micro-organisms. The present study was able to verify the H_2_O_2_ production of both *L. reuteri* strains. However, the method used was more qualitative than quantitative and did not allow the amount of H_2_O_2_ produced by the two strains to be determined. *Candida* species have developed mechanisms to detoxify reactive oxygen species (ROS), such as H_2_O_2_, in order to survive and evade the host immune system [39]. Ramírez-Quijas et al. [39] found that *C. albicans* was more susceptible to H_2_O_2_ compared with *C. glabrata, C. parapsilosis*, and especially *C. krusei* which was the most resistant of the four species. There is a good possibility that the non-*albicans Candida* species possess mechanisms other than those possessed by *C. albicans* to detoxify ROS. Accordingly, it has been proposed that the resistance of *C. krusei* and *C. parapsilosis* to H_2_O_2_ is due to the presence of an oxidase which is part of an alternative oxidative pathway [40].

It is important to stress that *in vitro* studies always have limitations, since they can never mimic the complex microbiota found in the oral cavity. In general, the effects of probiotic bacteria are highly strain specific, which was confirmed in this study. Even at strain level, significant differences in co-aggregation and growth inhibition abilities were found between the two *L. reuteri* strains. This indicates that probiotics as such cannot just be put in one barrel and that further *in vitro* and, more importantly, *in vivo* investigations are needed to increase the understanding on the role of probiotic supplements in the prevention and management of *Candida* infections in the oral cavity.

## Conclusions

The two probiotic *L. reuteri* strains exhibited antifungal properties against five of the six most common *Candida* species in the oral cavity. The findings clearly showed that some *Candida* species, particularly *C. krusei*, were not affected by the probiotic interference. The probiotic properties differed significantly between the two *Lactobacillus* strains. Thus, the understanding that the impact of probiotic supplements is strain and host specific was reconfirmed.
